# The validity and reliability of script concordance test in otolaryngology residency training

**Published:** 2016-04

**Authors:** KAMYAR IRAVANI, MITRA AMINI, AIDA DOOSTKAM, MAHNAZ DEHBOZORGIAN

**Affiliations:** 1 Otolaryngology Research Center, Department of Otolaryngology, Shiraz University of Medical Sciences, Shiraz, Iran;; 2 Quality Improvement in Clinical Education Research Center, Education Development Center, Shiraz University of Medical Sciences, Shiraz, Iran;; 3 Department of Pharmacology, Shiraz University of Medical Sciences, Shiraz, Iran

**Keywords:** Training, Otolaryngology, Validity, Reliability

## Abstract

**Introduction:**

The script concordance test (SCT) is one the best tools used to evaluate clinical reasoning in ill-defined clinical situations. The aim of this study was to demonstrate SCT application in otolaryngology residency training.

**Methods:**

A 20 item otolaryngology SCT containing 60 questions was administered to 26 otolaryngology residents. The test was prepared by two otolaryngologists familiar to medical education. These questions have been validated by otolaryngology experts. The panel consisted of 9 academic staff in the field of otolaryngology. Pearson correlation test was used to assess the reliability of the test.

**Results:**

The obtained mean scores were 68.4±5.8 (out of 100) for residents and 78.2±6.4(out of 100) for experts. There was a significant difference between the two scores (p<0.005). Cronbach’s alpha value was 0.80.

**Conclusion:**

The SCT is a reliable tool to evaluate clinical reasoning in otolaryngology residents. It should be included in otolaryngology residency training.

## Introduction

Clinical reasoning is one of the most important skills for physicians to solve ill-defined problems (1, 2). So, students and residents should be trained to have a strong clinical reasoning skill.

It makes the physicians choose the appropriate diagnostic tools and treatment options in various situations. Clinical reasoning is a process by which clinical data from a medical problem combined with the previous knowledge and experience of the physician are used to manage uncertain clinical situations. Clinical reasoning is an important factor for the physician’s competence. Medical education experts believe that clinical reasoning should be taught for medical students and residents. Several tests have been introduced to assess clinical reasoning including key feature (KF), script concordance (SCT), clinical reasoning problems (CRP), and comprehensive integrative puzzles (CIP). These tests are prepared and validated by experts in their fields (1).

In otolaryngology, like other clinical sciences, physicians challenge with new and alternative diagnostic and treatment options called ill-defined problems. These problems indicate that there is more than one solution existing for the problems (1).

According to cognitive psychology script theory, scripts are the networks of knowledge in our minds. Clinicians use scripts in judgment for diagnosis and treatment options in uncertain and ill-defined cases (3).

Scripts appear from the beginning of education in the medical school and are refined in postgraduate state with clinical experiences (4). Script concordance test (SCT) is one of the best tools to assess the clinical reasoning. It helps us together with other tools but cannot be replaced with them for clinical reasoning evaluation (5). Script concordance test constitutes a clinical situation (hypothesis) in domains of diagnosis, treatment or investigation in the first part. In the second part, the examinees were confronted with new information and asked to define the effect of new findings (information) on the hypothesis by a five point Likert scale. Scoring was the degree of concordance for responses with those of experts’ panel (6, 7).

There are a series of studies on validity and reliability of SCT in the fields of radiation oncology (1), urology (8), rheumatology (2), family medicine (9, 10), but no study has been done in the field of otolaryngology yet. The aim of this study was to define the usefulness of SCT on clinical reasoning in otolaryngology residents.

## Methods

The participants consisted of 26 senior (3^rd ^and 4^th^ year) residents in otolaryngology department of Shiraz University of Medical Sciences. Each examinee received information about the goals and format of examination before the test. A test sample was also demonstrated for examinees before the examination. Items and questions were constructed by two otolaryngologists familiar with medical education and also cooperation of Education and Development Center (EDC) in Shiraz University. Experts’ panel consisted of 9 board certified otolaryngologists in

Our department and another qualified center. They also participated in the exam for future comparison with the residents.

The test consisted of 20 items and 60 questions in the field of otolaryngology and has been designed for residency training. The items were taken from the prevalent cases in the field of otolaryngology. These cases incorporate the uncertainty on management and diagnosis (appendix 1).

***SCT scoring***

For scoring, we used the formula 1/ (1+x) where x is defined as the distance between the selected and correct answer (x ranged from 1 to 5 in a five – point Likert scale). This is an innovative method advised by Bland et al. and used by Amini et al. (5). It seems to be more accurate than other scoring methods (11).

The maximum score for an answer was 1, which was chosen by the majority of panels. The other panel choices had a partial credit. Average MCQ scores from4 annual and periodic examination were gathered to be compared with SCT scores to find any correlation.

Otolaryngology experts also validated the content of questions. They were not the same as those constructed for the exam. 

Test reliability was calculated by the Cronbach alpha coefficient. Good reliability is indicated when the coefficient is ≥0.80. Item analysis was done to detect the problematic questions. Data analysis was done by SPSS software, version 11.0. Item analysis was done by Whitney and Sabers method to detect problematic questions.  

***Case description*** *: a 50-year-old patient with stage II laryngeal carcinoma for whom radiotherapy was done. Three months after termination of radiotherapy, the patient was referred with tumor recurrence.*

**Apendix 1:** Example of an otolaryngology script concordance test

**If you were considering**	**And then you find**	**Your recommendation become:**
**Supracricoidlaryngectomy**	COPD	-2 -1 0 +1 +2
**Laser surgery**	Chronic bronchitis	-2 -1 0 +1 +2
**Total laryngectomy**	Thyroid cartilage involvement	-2 -1 0 +1 +2
***Scoring key: ***** -2:strongly contraindicated, -1:contraindicated 0:neither more or less indicated ( It doesn’t change your mind ) +1: indicated, +2: strongly indicated**		

## Results

26 residents and 9 experts participated in the study. Although there was no time limitation, all the participants completed the test in less than an hour. The mean scores and the score variability were 68.4±5.8 out of 100 (range=52.8-77.6) for residents and 78.2±6.4 out of 100 (range=62.3-91.7) for the panel. 

Item analysis was done. Then, two questions were excluded due to poor item difficulty (questions no. 3 and 11). An acceptable level of difficulty was between 0.30 to 0.80. Then, the final score was calculated. The result of item analysis is shown in Table 1.

**Table1:** Item difficulty level and item-total correlations

	q1	q2	q3	q4	q5	q6	q7	q8	q9	q10
**Item difficulty level**	0.52	0.60	0.15	0.44	0.57	0.49	0.43	0.40	0.49	0.42
**Item- total correlation**	0.46	0.46	0.12	0.30	0.33	0.31	0.30	0.42	0.40	0.30
	q11	q12	q13	q14	q15	q16	q17	q18	q19	q20
**Item difficulty level**	0.2	0.51	0.42	0.47	0.52	0.57	0.48	0.44	0.49	0.51
**Item- total correlation**	0.1	0.30	0.31	0.40	0.32	0.30	0.51	0.34	0.30	0.46

There was a significant difference between the mean score of residents and the panel (P<0.05). The Cronbach’s alpha coefficient was 0.80 for the reliability of the test. The correlation between SCT and MCQ scores is shown in Table 2. The correlation was significant at the 0.05 level (2-tailed).

**Table 2:** Correlation between MCQ and SCT scores

**Pearson correlation**	**MCQ**	**SCT**
**MCQ** **Sig. (2-tailed)**	1	0.458 0.019
**SCT** **Sig. (2-tailed)**	0.458 0.019	1

*correlation is significant at the 0.05. level (2-tailed)

All 26 residents and 9 experts signed an informed consent about the examination. All 26 residents were volunteer to take the exam. They were informed that the test was not mandatory and their scores were not used for any certification. 

## Discussion

Physicians need knowledge, experience and clinical reasoning to solve diagnostic and treatment problems. Script concordance test (SCT) is one of the best tools to evaluate the clinical reasoning ability. Clinical reasoning is based on data interpretation that needs correlation between previous and new data in an organized manner (7).

With increasing body of research from other centers, clinical reasoning tests including SCT may be applied in the future routinely.

In this study, we constructed a SCT for otolaryngology residents in the first time to assess its reliability and validity as a valuable tool in future. The test appeared reliable with Cronbach’s alpha coefficient value of 0.80. Other researchers have used the test in various fields in pre- and post-graduate students. Lambert et al. administered the test to radiation oncology students and residents with resulting Cronbach’s alpha of 0.90 (1). Mathieu et al. utilized online version of SCT for the 5th year medical students in rheumatology with Cronbach’s alpha value of 0.82 (2). Also, Lemay et al. used SCT in pediatric students and residents with acceptable reliability coefficient of 0.74 (12). Amini et al. used SCT for top-ranked medical students with a reliability coefficient of 0.78(5).

Experts’ panel had a higher level of experience and a higher mean score than residents in SCT scoring. This is compatible with the findings of other studies (1, 2). So, SCT may be used as a reflection of clinical experience in pre- and post-graduate medical groups, especially during residency training.

Caire et al. used SCT as a training tool for neurosurgery interns by applying it as a self-assessment examination that assessedthe clinical experience (13, 14). Humbert et al. also indicated that SCT may assess clinical experience in examinees.Their study was satisfactory with Cronbach’s alpha of 0.73, that is near to our study result (15).

There was a positive correlation between MCQ and SCT scores in our study. This may be due to good MCQ scores; the examinees had a well-organized knowledge for reasoning together with experience.

The major limitation of this study was lack of familiarity of the panel and examinees to the examination format in spite of pretest instruction. Other studies mentioned this problem (1, 2). Another limitation of our study and other similar researches is small cohort of participants and panels. Multicenter studies are needed for better assessment of the test.

## Conclusion

Our study showed that script concordance test is a reliable test to assess clinical reasoning in otolaryngology residents. It needs to provide additional data from multiple researches to conclude the usefulness of the test for widespread use in residency training. 
